# Therapeutic Effects of Two Different Molecular Weights of Orally Administered Hyaluronan, Both as Monotherapy and in Combination with Methotrexate in a Rat Model of Arthritis †

**DOI:** 10.3390/ijms26093958

**Published:** 2025-04-22

**Authors:** Sasan Khademnematolahi, Silvester Ponist, Karol Svik, Frantisek Drafi, Lukas Slovak, Jana Muchova, Elisabeth Louise Mindang, Waqar Ahmad, Katarina Bauerova

**Affiliations:** 1 Centre of Experimental Medicine SAS, Institute of Experimental Pharmacology and Toxicology, 841 04 Bratislava, Slovakia; exfasasa@savba.sk (S.K.); silvester.ponist@savba.sk (S.P.); exfakasv@savba.sk (K.S.); exfadrfr@savba.sk (F.D.); exfawaqa@savba.sk (W.A.); 2 Faculty of Natural Sciences, Comenius University, 814 99 Bratislava, Slovakia; 3 State Institute for Drug Control, 825 08 Bratislava, Slovakia; lukas.slovak@sukl.sk; 4 Institute of Medical Chemistry, Biochemistry and Clinical Biochemistry, Faculty of Medicine, Comenius University, 811 08 Bratislava, Slovakia; jana.muchova@fmed.uniba.sk; 5 Department of Animal Biology and Physiology, Faculty of Science, University of Yaounde I, Yaoundé P.O. Box 337, Cameroon; ndjenguelouiseelisabeth@yahoo.com

**Keywords:** hyaluronan, arthritis, methotrexate, Lewis rats, oxidative stress, inflammation

## Abstract

Rheumatoid arthritis (RA) is a chronic autoimmune disease characterized by joint inflammation and systemic involvement. This study investigates the therapeutic potential of oral hyaluronan (HA) with different molecular weights (SHA: 0.99 MDa and VHA: 1.73 MDa) as monotherapy and in combination with methotrexate (MTX) in a preclinical adjuvant arthritis (AA) model in Lewis rats. The aim was to evaluate the impact of these treatments on biometric, inflammatory, and oxidative stress parameters. The preliminary study tested two doses of HA (0.5 mg/kg and 5 mg/kg), and the pivotal study focused on the combination of 0.5 mg/kg HA with 0.3 mg/kg MTX. Based on our experimental findings on combined therapy, the MTX + SHA combination demonstrated superior efficacy compared to MTX + VHA and MTX monotherapy. Specifically, the MTX + SHA regimen significantly promoted weight gain and reduced hind-paw volume in all monitored experimental days. This treatment markedly reduced plasmatic IL-17A levels (day 21) and GGT activity in both the spleen and joints (day 28), showing the most pronounced effects among all groups, including the MTX monotherapy group. The MTX + VHA combination showed a therapeutic response comparable to MTX alone, indicating no additional benefit. These findings suggest a superior efficacy of the MTX + SHA combination in comparison to other studied treatments. The overall efficacy can be ranked as: MTX ≈ MTX + VHA < MTX + SHA.

## 1. Introduction

Rheumatoid arthritis (RA) is a heterogeneous, inflammatory, chronic autoimmune disease that starts to affect small joints and then progresses to bigger joints and to causing inflammation, morning joint stiffness, and pain [1,2]. RA is most common in people who are 30–50 years of age and predominantly affects women [3,4]. Many factors, including an increase in reactive oxygen species (ROS) production, systemic inflammation, and epigenetic modifications are involved in the development of chronic autoimmune diseases such as RA [4,5]. Over the past decade, nonsteroidal anti-inflammatory drugs (NSAIDs), as well as disease-modifying antirheumatic drugs (DMARDs) such as methotrexate (MTX), have enhanced the success of RA management [6,7]. Methotrexate, one of the most commonly prescribed DMARDs, is widely regarded as the gold standard for RA [8]. Both in monotherapy and in combination therapy, MTX has demonstrated efficacy and a tolerable long-term safety profile. However, our studies and other authors have shown that combination treatments of MTX with supplements or natural extracts have greater efficacy than single treatment in the management of experimental arthritis [9,10,11,12,13,14,15]. In our complex study, we have used adjuvant arthritis (AA), a well-established model of RA that is suitable for preclinical research, as we have demonstrated in our previous experiments [9,10,13,14,15,16]. AA is an autoimmune joint inflammation model induced by an intradermal injection of heat-inactivated *Mycobacterium butyricum* in Freund’s incomplete adjuvant. In Lewis rats, arthritis develops following a single subcutaneous injection of prepared complete Freund’s adjuvant (CFA), which elicits a strong Th1/Th17-driven immune response. Due to their genetic susceptibility, these rats experience a self-sustaining inflammatory process, with peak inflammation occurring around 2–3 weeks post-injection, followed by natural resolution after 4–5 weeks. Days 14, 21, and 28 were chosen to reflect key stages of arthritis’ progression onset, peak inflammation, and chronic phase. This high reproducibility makes AA one of the most widely used models in RA research [17]. Weight loss, swelling of joints, generation of inflammatory cytokines, and activation of the immune system are all symptoms of both RA and AA [17,18].

Hyaluronan (HA) is a naturally occurring carbohydrate, more precisely a mucopolysaccharide, found in all creatures. It can bind thousands of carbohydrates to water, giving it a stiff, viscous characteristic comparable to jelly. HA is a polysaccharide with a poly repeating disaccharide structure [(1→4)-(2-Acetamido-2-deoxy-D-gluco)-(1→3)-D-glucuronoglycan] [19]. HA is most abundant in the extracellular and pericellular matrix. The physicochemical properties of HA are crucial to its chondroprotective effects and explain its protective effects on articular cartilage [20]. HA has protective effects on cartilage degradation in experimentally induced osteoarthritis. It is known that cartilage contains endogenous hyaluronan [21,22]. Endogenous HA modifies immune cell behavior, increases chondrocyte HA and synthesis of proteoglycan, and decreases the generation and activity of matrix metalloproteinases and inflammatory mediators. Scavenging ROS, inhibiting immune complex adhesion to granulocytes and inhibiting immune cell motility and aggregation, are examples of how these functions are demonstrated [23]. HA is not only a structural component but also controls cell migration, proliferation, and differentiation via interacting with receptors on the cell surface [24,25]. How the molecular weight of HA affects its biological activity was investigated recently in several studies [26,27]. Medium- and high-weight HA molecules from 50 to 1800 kDa increased the healing process, but the very small-weight HA molecules (around 6 kDa) demonstrated inflammatory action [28,29]. Depending on their molecular weight, HA molecules could be classified into four groups: low-molecular-weight HA (10–250 kDa), medium-molecular-weight HA (250–1000 kDa), high-molecular-weight HA, (>1000 kDa), and very high-molecular-weight HA (>6000 kDa) [30].

This study focuses on two specific molecular weight variants of HA: SHA (0.99 MDa) and VHA (1.73 MDa). SHA falls within the high-molecular-weight (HMW) HA category (>1000 kDa), but it is very close to the upper limit of medium molecular weight, and VHA can be referred to as high-molecular-weight HA. These variants were selected based on their potential to modulate inflammation and oxidative stress in an arthritis model.

In standard clinical practice, injections of HA drugs into affected joints have been applied as a therapeutic intervention for osteoarthritis. These therapeutic approaches are aimed to renew the depleted amounts of HA in the joints, reduce inflammation, improve lubrication of joints, and provide symptomatic ease. A variety of clinical studies have shown that HA intra-articular injection is effective in reducing pain, improving joint function, and postponing the need for surgery in patients with osteoarthritis and RA [31,32]. However, the effects of oral administration of HA as monotherapy or in combination with standard disease-modifying antirheumatic drugs have not been determined yet. The aim of this experiment was to find out whether two different high molecular weights of hyaluronan (HMW-HA) (1730 kDa and 990 kDa) molecules will have a therapeutic effect in experimental arthritis when administered alone and in combination with methotrexate.

## 2. Results

### 2.1. Biometric Parameters Obtained in the Preliminary Experiment

The preliminary experiment, a study designed to determine the optimal dosage based on hyaluronan monotherapy, paved the way for the next combination therapy investigation. The key biometric markers were measured together with selected indicators of oxidative stress in erythrocytes and plasma.

#### 2.1.1. The Change in Animal Body Weight During the Preliminary Experiment

**Figure 1:**
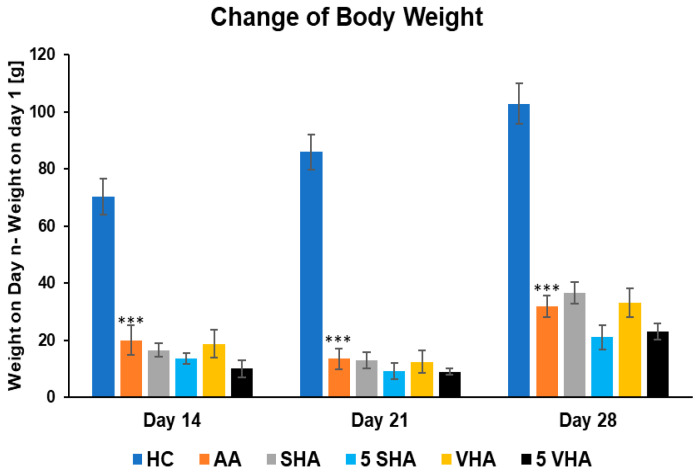
The change in animal body weight was measured on days 14, 21, and 28 of the preliminary experiment. The experimental animals were allocated into the following groups: HC—healthy control; AA—adjuvant arthritis; SHA—AA administered orally with hyaluronan (HA) of molecular weight 0.99 MDa at a dose of 0.5 mg/kg body weight/daily; 5 SHA—AA administered orally with HA of molecular weight 0.99 MDa at a dose of 5 mg/kg body weight/daily; VHA—AA administered orally with HA of molecular weight 1.73 MDa at a dose of 0.5 mg/kg body weight/daily; 5 VHA—AA administered orally with HA of molecular weight 1.73 MDa at a dose of 5 mg/kg body weight/daily. Results are shown as a mean ± standard error of the mean (S.E.M). ANOVA was conducted to assess the statistical significance concerning independent variables. The symbols indicating the significant change are as follows: *** *p* ≤ 0.001 AA vs. HC.

Monitoring body weight in arthritic rats provides insight into systemic disease impact. In RA models like AA, animals typically lose weight due to inflammation-induced cachexia (muscle wasting and anorexia). This weight loss is driven by proinflammatory cytokines (e.g., TNF-α) and reflects the severity of systemic inflammation. Thus, change in body weight has been determined to act as an overall health indicator, with reduced weight gain signaling active RA pathology [33]. Figure 1 shows the changes in body weight throughout the period of treatment during the preliminary experiment. Following experimental days 14, 21, and 28, the change in body weight (ChBW) parameter was considerably lower in the untreated AA animal group compared to the healthy control (HC) group (*** *p* ≤ 0.001 AA vs. HC). None of the other treatments demonstrated a significant effect.

#### 2.1.2. The Change in Hind-Paw Volume During the Preliminary Experiment

**Figure 2:**
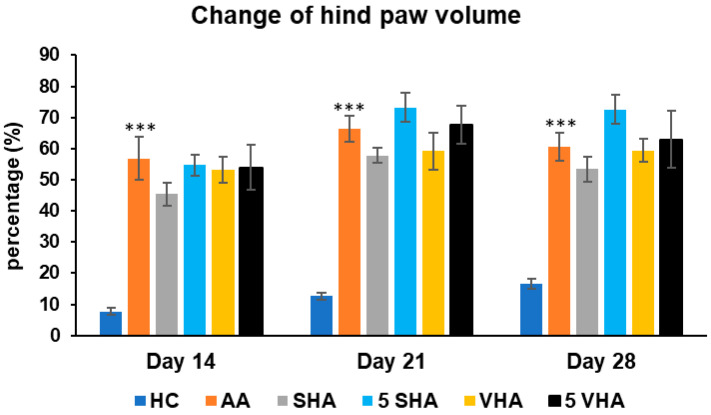
Change in hind-paw volume was measured on days 14, 21, and 28 of the preliminary experiment. The experimental groups are the same as described in Figure 1. Results are shown as a mean ± standard error of the mean (S.E.M). ANOVA was conducted to assess the statistical significance concerning independent variables. The symbols indicating the significant change are as follows: *** *p* ≤ 0.001 AA vs. HC.

Hind-paw volume is a key indicator of joint inflammation and swelling in AA, mimicking RA. Increased paw volume reflects synovial inflammation, immune cell infiltration, and edema, primarily driven by proinflammatory cytokines like TNF-α, IL-1β and IL-17A. Measuring paw volume helps assess disease progression and treatment effectiveness in reducing inflammation [34]. The results presented in Figure 2 indicate a significant difference in the hind-paw volume observed in the preliminary experiment. The change in the hind-paw volume (CHPV) was measured on days 14, 21, and 28. AA increased the HPV significantly as compared to HC on days 14, 21, and 28 of the experiment (*** *p* ≤ 0.001 AA vs. HC). None of the other treatments demonstrated a significant effect.

### 2.2. The Activity of Antioxidant Enzymes in Erythrocytes and Plasmatic Level of Lipoperoxides in the PreliminaryExperiment

#### 2.2.1. The Activity of Superoxide Dismutase in Erythrocytes

**Figure 3:**
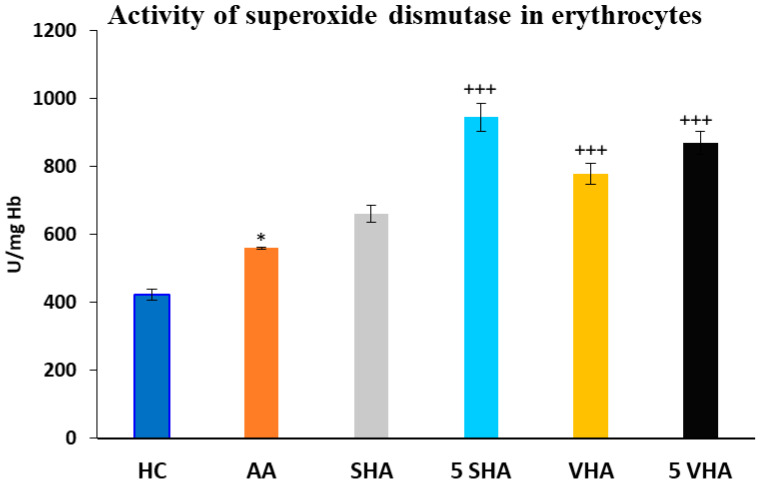
Activity of superoxide dismutase (SOD) in erythrocytes on the 28th day. The experimental groups are the same as described in Figure 1. Results are shown as a mean ± standard error of the mean (S.E.M). ANOVA was conducted to assess the statistical significance concerning independent variables. The symbols indicating the significant change are as follows: * *p* ≤ 0.05 AA vs. HC; +++ *p* ≤ 0.001 treated groups vs. AA.

Antioxidant enzymes like superoxide dismutase (SOD) and glutathione peroxidase (GPx) play a vital role in protecting erythrocytes from oxidative damage. In RA and AA, elevated oxidative stress contributes to inflammation and joint damage. Measuring SOD and GPx activity in erythrocytes helps assess systemic oxidative balance, disease progression, and the potential benefits of antioxidant-based treatments [35]. Superoxide dismutase (SOD) activity was assessed on the 28th day of the experiment in erythrocytes. The results depicted in Figure 3 revealed a significant increase in superoxide dismutase activity compared to the HC experimental group (* *p* ≤ 0.05 AA vs. HC). As compared to the AA group of animals, the activity of SOD was significantly increased by both molecular weights of HA monotherapy at a dose of 5 mg/kg body weight/daily and in the VHA group with a dose of 0.5 mg/kg body weight/daily (AA-5 VHA: +++ *p* ≤ 0.001 vs. AA; AA-VHA: +++ *p* < 0.001 vs. AA; AA-5 SHA: +++ *p* ≤ 0.001 vs. AA) (Figure 3).

#### 2.2.2. The Activity of Glutathione Peroxidase in Erythrocytes

**Figure 4:**
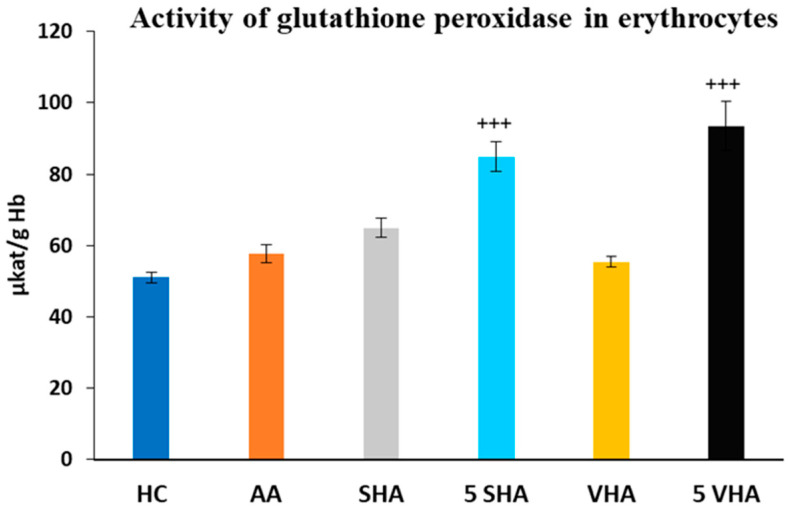
Activity of glutathione peroxidase (GPx) in erythrocytes on the 28th day. The experimental groups are the same as described in Figure 1. Results are shown as a mean ± standard error of the mean (S.E.M). ANOVA was conducted to assess the statistical significance concerning independent variables. The symbols indicating the significant change are as follows: +++ *p* ≤ 0.001 treated groups vs. AA.

Glutathione peroxidase (GPx) activity was assessed on the 28th day of the experiment in erythrocytes. The activity of GPx, as depicted in Figure 4, showed no significant difference compared to the HC experimental group. Further, the results depicted in Figure 4 revealed that the activity of GPx, in comparison with the AA group of animals, was significantly increased by both molecular weights of HA monotherapy at a dose of 5 mg/kg body weight/daily. VHA and SHA did not demonstrate any significant effect (AA-5 VHA: +++ *p* ≤ 0.001 vs. AA; AA-5 SHA: +++ *p* ≤ 0.001 vs. AA (Figure 4).

#### 2.2.3. Concentration of Lipoperoxides in Plasma

**Figure 5:**
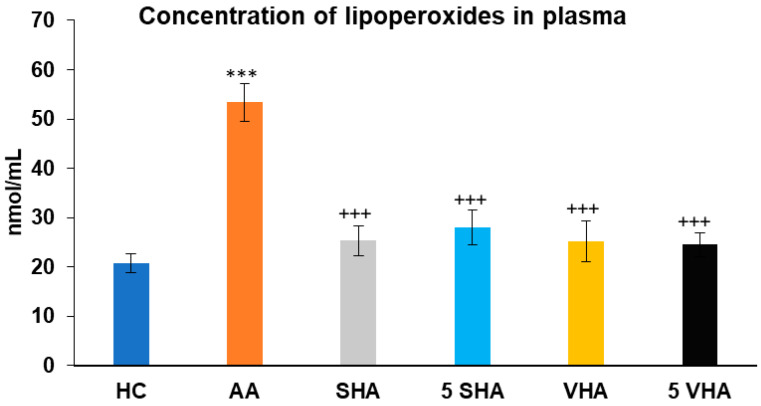
Concentration of lipoperoxides (LPxs) in plasma on day 28. The experimental groups are the same as described in Figure 1. Results are shown as a mean ± standard error of the mean (S.E.M). ANOVA was conducted to assess the statistical significance concerning independent variables. The symbols indicating the significant change are as follows: *** *p* ≤ 0.001 AA vs. HC; +++ *p* ≤ 0.001 treated groups vs. AA.

Lipoperoxides (LPxs) are markers of lipid peroxidation, indicating oxidative damage to cell membranes. Elevated LPx plasmatic levels reflect increased oxidative damage in RA and AA, making it a valuable indicator of disease severity and the effectiveness of antioxidant properties [36]. Lipoperoxide (LPx) concentration was assessed on the 28th day of the experiment in plasma. The results presented in Figure 5 showed that the concentration of LPx significantly increased in comparison to that of the HC experimental group (*** *p* ≤ 0.001 AA vs. HC). When compared to the AA group of animals, the activity of LPx in plasma was significantly decreased by all tested HA therapies (AA-SHA: +++ *p* ≤ 0.001 vs. AA; AA-VHA: +++ *p* < 0.001 vs. AA; AA-5 VHA: +++ *p* ≤ 0.001 vs. AA; AA-5 SHA: +++ *p* ≤ 0.001 vs. AA) (Figure 5).

### 2.3. Biometric Parameters from Combination Therapy Experiment—The Pivotal Study

All biometric parameters were assessed on days 14, 21, and 28 of the experiment in order to monitor the development of arthritis and the state of health of the animals.

#### 2.3.1. The Change in Animal Body Weight

**Figure 6:**
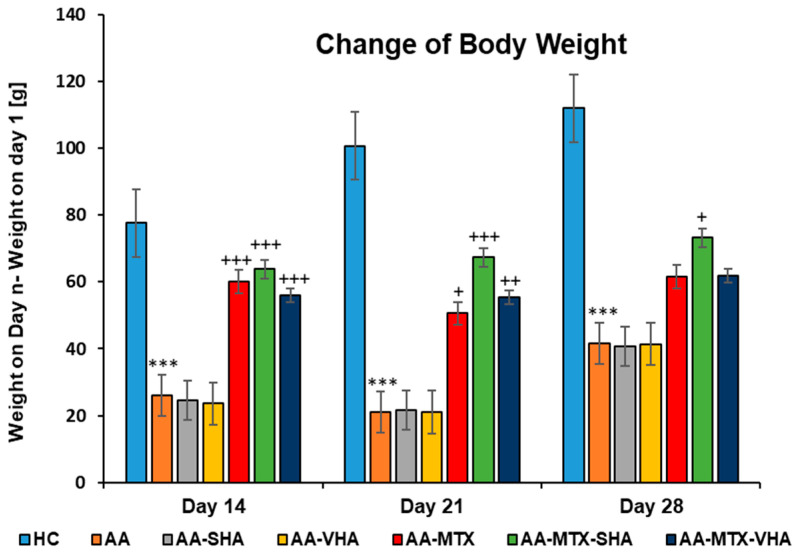
The change in animal body weight was measured on days 14, 21, and 28 of the experiment. The experimental animals were allocated into the following groups: HC—healthy control; AA—adjuvant arthritis; AA-SHA—AA administered orally with HA of molecular weight 0.99 MDa at a dose of 0.5 mg/kg body weight/daily; AA-VHA—AA administered orally with HA of molecular weight 1.73 MDa at a dose of 0.5 mg/kg body weight/daily; AA-MTX—AA administered orally with MTX at a dose of 0.3 mg/kg two times per week; AA-MTX-SHA—administered orally with HA of molecular weight 0.99 MDa at a dose of 0.5 mg/kg body weight/daily in combination with MTX 0.3 mg/kg two times per week; AA-MTX-VHA—administered orally with HA of molecular weight 1.73 MDa at a dose of 0.5 mg/kg body weight/daily in combination with MTX at a dose of 0.3 mg/kg two times per week. Results are shown as a mean ± standard error of the mean (S.E.M). ANOVA was conducted to assess the statistical significance concerning independent variables. The symbols indicating the significant change are as follows: *** *p* ≤ 0.001 AA vs. HC; +++ *p* ≤ 0.001, ++ *p* < 0.01 treated groups vs. AA, + *p* < 0.05 treated groups vs. AA.

Figure 6 shows the change in body weight throughout the period of treatment. Following experimental days 14, 21, and 28, the change in body weight (ChBW) parameter was considerably lower in the untreated AA animal group compared to the healthy control (HC) group (*** *p* ≤ 0.001 AA vs. HC). In comparison to the AA group, the results showed that the body weight increased in animals in the methotrexate (MTX) group (+++ *p* ≤ 0.001, day 14; + *p* ≤ 0.05, day 21 vs. AA) and the MTX group in combination with the HA—both molecular weights of HA in combination with MTX significantly increased the ChBW on day 14 (AA-MTX-SHA, +++ *p* ≤ 0.001 vs. AA and AA-MTX-VHA, +++ *p* ≤ 0.001 vs. AA) and day 21 (AA-MTX-SHA, +++ *p* ≤ 0.001 vs. AA and AA-MTX-VHA, ++ *p* ≤ 0.01 vs. AA). On day 28, only the treated AA-MTX-SHA group showed a significant increase in body weight in comparison to the AA group (AA-MTX-SHA, + *p* ≤ 0.05 vs. AA.) (Figure 6).

#### 2.3.2. The Change in Hind-Paw Volume of Experimental Animals

**Figure 7:**
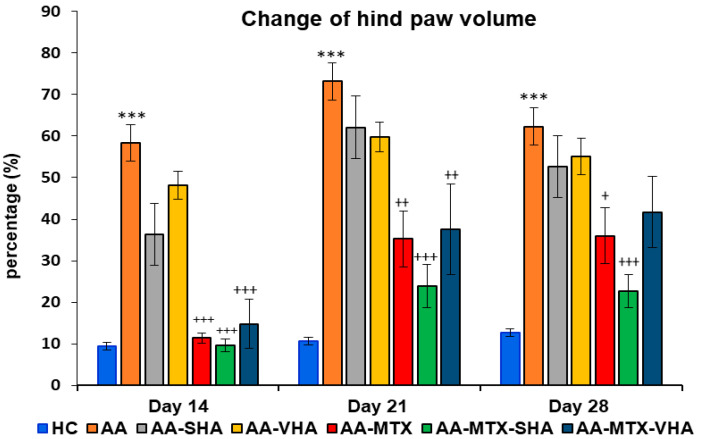
Change in hind-paw volume was evaluated on days 14, 21, and 28 of the experiment. The experimental groups are the same as described in Figure 6. Results are shown as a mean ± standard error of the mean (S.E.M). ANOVA was conducted to assess the statistical significance concerning independent variables. The symbols indicating the significant change are as follows: *** *p* ≤ 0.001 AA vs. HC; +++ *p* ≤ 0.001, ++ *p* < 0.01 and + *p* < 0.05 treated groups vs. AA.

The results depicted in Figure 7 demonstrate a significant difference in hind-paw volume. The change in hind-paw volume (CHPV) was evaluated on days 14, 21, and 28. AA significantly increased the HPV in comparison to HC on days 14, 21, and 28 of the experiment (*** *p* ≤ 0.001 AA vs. HC). In comparison to the untreated AA group, methotrexate applied in monotherapy and in combination with HA in both molecular weights (AA-MTX-SHA and AA-MTX-VHA) significantly decreased the hind-paw volume on day 14 (AA-MTX: +++ *p* ≤ 0.001 vs. AA; AA-MTX-SHA: +++ *p* ≤ 0.001 vs. AA; AA-MTX-VHA: +++ *p* ≤ 0.001 vs. AA), day 21 (AA-MTX: ++ *p* ≤ 0.01 vs. AA; AA-MTX-SHA: +++ *p* ≤ 0.001 vs. AA; AA-MTX-VHA: ++ *p* ≤ 0.01 vs. AA), and day 28 (AA-MTX: + *p* ≤ 0.05 vs. AA; AA-MTX-SHA: +++ *p* ≤ 0.001 vs. AA). AA-MTX-VHA was not significantly effective in changing HPV on day 28 (Figure 7), similar to its effect on BW, where significance was not also achieved (Figure 6).

### 2.4. The Activity of Gamma-Glutamyl Transferase in the Joint

Gamma-glutamyl transferase (GGT) activity is measured in joint and spleen tissues as a marker of tissue oxidative stress (OS) and inflammation. GGT is an enzyme involved in glutathione metabolism, and its activity rises under conditions of OS, reflecting the turnover of antioxidants. In RA, increased GGT in inflamed tissues has pathological significance. GGT acts as a bone-resorbing factor that promotes osteoclast formation, contributing to bone and joint destruction. Thus, the basis for determining GGT is to assess local oxidative/inflammatory burden, and its elevation is directly related to the joint damage characteristic of RA pathology [37].

**Figure 8:**
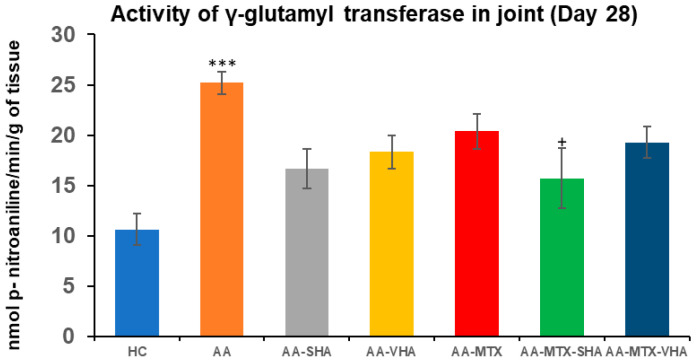
The gamma-glutamyl transferase (GGT) activity in the joint on the 28th day. The gamma-glutamyl transferase activity was measured on the 28th day of the experiment. The experimental groups are the same as described in Figure 6. Results are shown as a mean ± standard error of the mean (S.E.M). ANOVA was conducted to assess the statistical significance concerning independent variables. The symbols indicating the significant change are as follows: *** *p* ≤ 0.001 AA vs. HC and + *p* < 0.05 treated group vs. AA.

GGT activity was assessed on the 28th day of the experiment in the tissue homogenate of the hind-paw joint. The results depicted in Figure 8 showed that the activity of GGT in the joints significantly increased in comparison to the HC experimental group (*** *p* ≤ 0.001 AA vs. HC). In comparison to the AA group of animals, the activity of GGT in the joints was significantly reduced by SHA combination therapy (AA-MTX-SHA, + *p* ≤ 0.05 vs. AA). None of the other treatments demonstrated a significant effect (Figure 8).

### 2.5. The Activity of Gamma-Glutamyl Transferase in the Spleen

**Figure 9:**
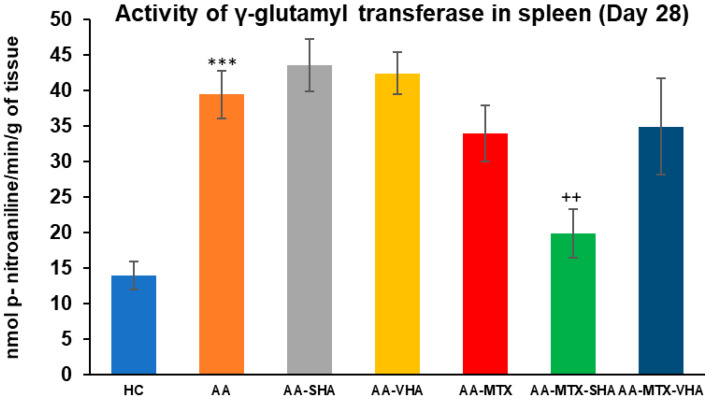
The gamma-glutamyl transferase (GGT) activity in the joint on the 28th day. The gamma-glutamyl transferase activity was measured on the 28th day of the experiment. The experimental groups are the same as described in Figure 6. Results are shown as a mean ± standard error of the mean (S.E.M). ANOVA was conducted to assess the statistical significance concerning independent variables. The symbols indicating the significant change are as follows: *** *p* ≤ 0.001 AA vs. HC and ++ *p* < 0.01 treated group vs. AA.

The gamma-glutamyl transferase (GGT) activity was assessed on the 28th day of the experiment in the tissue homogenate of the spleen. The results depicted in Figure 9 showed that the activity of GGT in the spleen significantly increased in comparison to the HC experimental group (*** *p* ≤ 0.001 AA vs. HC). In comparison to the AA group of animals, the GGT activity in the spleen was significantly lowered by SHA combination therapy (AA-MTX-SHA, ++ *p* ≤ 0.01 vs. AA). None of the other treatments demonstrated a significant effect; these results were similar to those of the assessment of joint tissue (Figure 9).

### 2.6. Levels of Interleukin 17A Measured in Plasma

IL-17A levels are measured to evaluate the systemic inflammatory cytokine response in RA. IL-17A is a proinflammatory cytokine produced by Th17 cells, known to play a significant role in RA pathogenesis. It stimulates synovial fibroblasts, macrophages, and chondrocytes to release other inflammatory mediators (like IL-6, IL-1, and TNF-alpha), thereby exacerbating inflammation and joint damage [38]. RA patients and arthritic animals often have elevated IL-17A, correlating with disease severity. Measuring IL-17A provides insight into the immune-mediated aspect of the disease, linking the experimental readout to the cytokine-driven pathology of RA.

**Figure 10:**
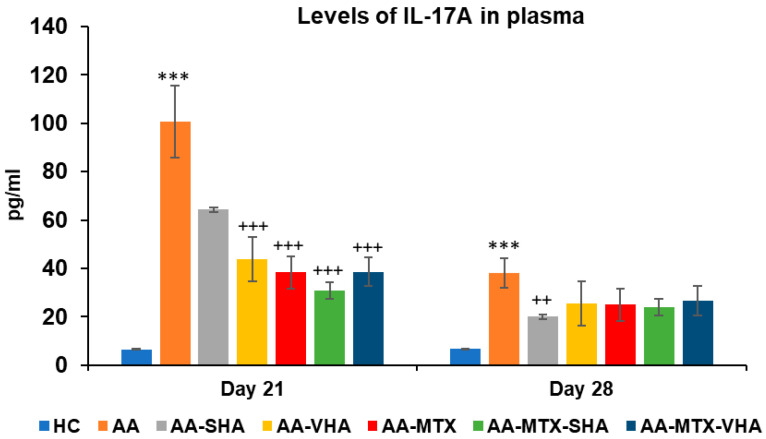
Plasmatic levels of IL-17A on days 21 and 28 of the experiment. The experimental groups are the same as described in Figure 6. Results are shown as a mean ± standard error of the mean (S.E.M). ANOVA was conducted to assess the statistical significance concerning independent variables. The symbols indicating the significant change are as follows: *** *p* ≤ 0.001 AA vs. HC; +++ *p* ≤ 0.001 and ++ *p* < 0.01 treated groups vs. AA.

Figure 10 represents the plasmatic level of interleukin 17A measured during day 21 and 28 of the experiment. The results demonstrate that AA significantly elevated the plasmatic level of interleukin 17A (IL-17A) in comparison to the HC group (*** *p* ≤ 0.001 AA vs. HC) on both monitored days. Monotherapies of AA-MTX and AA-VHA and combination of AA-MTX-SHA and AA-MTX-VHA showed significantly reduced level of plasmatic IL-17A compared to untreated AA on day 21 (AA-VHA: +++ *p* ≤ 0.001 vs. AA; AA-MTX: +++ *p* ≤ 0.001 vs. AA; AA-MTX-SHA: +++ *p* ≤ 0.001 vs. AA; AA-MTX-VHA: +++ *p* ≤ 0.001 vs. AA). On the 28th day, monotherapy with AA-SHA demonstrated a significantly reduced level of plasmatic compared to untreated AA (AA-SHA: ++ *p* ≤ 0.01 vs. AA). Other treatments were not significantly effective; however, they decreased the plasmatic level of the cytokine (Figure 10).

## 3. Discussion

Our approach to pharmacotherapy of rheumatoid arthritis (RA) is to find new combinations of MTX administered in subtherapeutic dose with natural substances with low toxicity to minimize the potential adverse effects of MTX. For this purpose, we use the well-established animal model of human RA, adjuvant arthritis (AA). AA was induced in Lewis rats, choosing only males, to ensure the reliability of our findings [39]. One of the promising RA treatment approaches could be the combination of MTX with two high molecular weights of hyaluronan (0.99 MDa and 1.73 MDa). In the preliminary experiment, we studied two doses of hyaluronan (HA) molecules: 0.5 mg/kg and 5 mg/kg. Both doses of VHA (1.73 MDa) and the higher dose of SHA (0.99 MDa) significantly changed the activity of SOD (Figure 3) in erythrocytes. The concentration of lipid peroxides in plasma (Figure 5) was significantly reduced by both doses of SHA and VHA. Since the lower dose of VHA and SHA was able to create a significant change in most of the relevant parameters, we have selected the lower dose for the combination treatment with MTX.

Meanwhile, there has been increasing research in recent years in the field of the anti-inflammatory and immunomodulatory effects of natural products, which have the potential to manage autoimmune conditions like RA [40,41]. Recent clinical trials have demonstrated that orally administered hyaluronan (HA) significantly alleviates knee pain, stiffness, and functional limitations in patients with osteoarthritis, highlighting its therapeutic potential [42]. Albano and his colleagues (2016) suggested that HMW-HA may be a better co-adjuvant than medium-molecular-weight hyaluronan (MMW-HA) for treating IL-17A-mediated nasal inflammation and oxidation. HMW-HA may regulate epithelial cell ROS, NOX-4, and IL-8 generation during nasal inflammation. Further, Albano et al. (2016) found, in his in vitro model, that HMW-HA may limit oxidative stress and inflammation by inhibiting the ERK1/2 intracellular signal pathway, which regulates NF-kB transcription [43].

The bioavailability of orally administered HA is much lower than intravenous [44]. Following oral ingestion, HA is absorbed through the intestinal pathway, with absorption and transport rates being influenced by its molecular weight [45]. The CD44 antigen, a transmembrane glycoprotein type 1, serves as the primary receptor for hyaluronan. It is found on the cell membranes of nearly all human cells [45]. HA (molecular weight 900 kDa; Hyabest^®^) was orally administered to MRL-lpr/lpr mice, a model of Th-1-type autoimmune disease. Upon ingestion, HA binds to the toll-like receptor-4 (TLR-4) in the intestine. Cytokine array analysis revealed that HA stimulates the production of interleukin-10. Additionally, DNA analysis of large intestine tissue demonstrated that HA increases the expression of suppressor of cytokine signaling 3 (SOCS3) while reducing pleiotrophin expression. These findings suggest that HA’s interaction with TLR-4 enhances IL-10 and SOCS3 expression while inhibiting pleiotrophin expression, contributing to its anti-inflammatory effects in mouse arthritis [46].

Although the reasons for differences in the activity of SHA and VHA are not known, they may include their molecular weight, which could considerably influence pharmacokinetic parameters, such as absorption, metabolic behavior, and enzymatic stability [47,48] and, furthermore, substantially affect interactions with receptors such as CD44 and TLR4 [49]. The molecular weight of SHA hyaluronan used in this study is 990 kDa, which is very close to 900 kDa Hyabest^®^. Thus, we suppose a similar mechanism of action as that of Hyabest^®^. In clinical trials with patients suffering from osteoarthritis, the authors found improvements in pain relief, joint stiffness, and WOMAC score after oral administration of HA [50]. Therefore, we assessed the potential antiarthritic and anti-inflammatory effects of hyaluronan in AA after its oral administration. We examined its capacity to significantly influence biometric parameters such as body weight change and hind-paw volume (see Section 2.1 and Section 2.3: Biometric parameters). It is important to recognize that AA is also a model for inflammatory cachexia, a condition that becomes increasingly evident over time [33]. The combination of 0.99 MDa molecular weight of HA with MTX significantly increased the weight gain in AA animals on day 28, whereas MTX monotherapy did not demonstrate any significant results (Figure 6). In our experiments, we were the first to demonstrate an increase in the weight of animals with AA by orally administering a combination of HA and MTX (Figure 6, day 28). On day 21 and 28 of the experiment, combination therapy of SHA and MTX significantly reduced hind-paw volume; however, there was no significant difference between MTX in monotherapy and the combination. Only the reduction in hind-paw swelling by AA-MTX-SHA, expressed as the average of this group, and concerning all monitored days, was more efficient than MTX monotherapy (Figure 7). HA monotherapy did not yield significant effects on biometric parameters. However, its combination with methotrexate may enhance therapeutic outcomes (Figure 6 and Figure 7).

AA is used as an experimental model of polyarthritis. Due to its systemic inflammatory processes, it induces pathological changes in various tissues, such as splenomegaly, thereby enabling the study of both joint and extra-articular manifestations of the disease [51]. Thus, in order to learn more about the effects of HA and MTX administration alone and in combination, we also assessed the GGT activity in the spleen and joint. The spleen GGT activity was determined in accordance with our earlier findings [52]. GGT is a non-specific measure of oxidative stress and inflammation, and its activity is intimately linked to the organism’s total antioxidant state, making it a crucial part of inflammatory processes. On day 28, HA alone did not affect the GGT activity in the joint and spleen (Figure 8 and Figure 9). The combined therapy of MTX-SHA was the only therapy which significantly decreased the GGT activity in both the spleen and joint, demonstrating its superiority when compared with MTX monotherapy or AA-MTX-VHA. It has been discovered that GGT stimulates the expression of RANKL on osteoblasts. Apart from the powerful bone-resorbing agents such as TNF-α, IL-1, and IL-6, GGT may also have a role in the pathological expression of RANKL during arthritis. Crucially, GGT affects the progenitors of osteoclasts as well [37]. Therefore, the therapeutic effect of the HA and MTX combination might be due to its ability to reduce GGT enzymatic activity in the joint and spleen, which subsequently decreases the expression of RANKL, TNF-α, IL-1, and IL-6.

The level of IL-17A in plasma on day 21 was shown to be significantly reduced by both tested combinations and MTX (Figure 10). Further, in our study, SHA also significantly decreased IL-17A levels when used as monotherapy at day 28 (Figure 10). This outcome is consistent with the recent scientific literature. Reduction in IL17 levels indicates that the treatment reduces Th17-related reinforcement of inflammation. SHA like HA, is likely to act on TLR2 and TLR4 receptors [53,54].

From our experiment with combined therapy, we can conclude that the MTX + SHA combination was the only one to significantly improve weight gain on day 28 and to reduce GGT activity in the spleen and joint; therefore, it is more effective than the MTX + VHA combination and MTX monotherapy. The combination of MTX + VHA was approximately at the same level of efficacy as MTX monotherapy (efficacy ranking: MTX ≈ MTX + VHA < MTX + SHA) (Figure 6, Figure 7, Figure 8 and Figure 9).

The systemic inflammation characteristic of RA extends beyond joints, affecting blood circulation and exposing red blood cells (RBCs) to inflammatory mediators that can impair their structure and function. Red blood cells in RA patients may be particularly vulnerable to oxidative stress (OS) due to chronic inflammation. Olumuyiwa-Akeredolu et al. (2017) reported structural and functional changes in RBCs, linking RA to impaired membrane integrity, altered blood flow dynamics, and increased cardiovascular risk [55]. Cimen et al. (2000) found that RA patients exhibited increased levels of OS markers, including elevated SOD, xanthine oxidase, and malondialdehyde, while GPx and CAT enzyme activities remained unchanged compared to healthy individuals [56]. Additionally, concerning the influence of OS, excess NO in RA promotes oxidative damage, particularly joint deterioration, making it a key therapeutic target [57].

Our findings in AA rats revealed increased SOD activity, unchanged GPx levels, and elevated plasma lipid peroxidation [58], aligning with Sarban et al. (2005), who reported similar oxidative stress patterns in RA patients [59]. In our experiment using AA, both SHA and VHA effectively reduced plasma lipid peroxidation, indicating their antioxidant potential. VHA demonstrated strong independent activity by increasing SOD and GPx activity without MTX, while SHA showed enhanced effects in combination therapy. Our study is the first to examine the effects of two hyaluronan molecules (0.99 MDa and 1.73 MDa) on the activity of SOD in erythrocytes from AA rats. The observed increase in erythrocyte SOD activity may be linked to CD44 receptor activation [60], reflecting complex HA–cell interactions. Overall, both HA molecular weights contribute to systemic OS reduction, supporting their therapeutic value in RA.

Taking into account all the results that we observed in both experiments with AA, we can predict the therapeutic potential of oral administration of high-molecular-weight HA (particularly 0.99 MDa molecular weight) for patients with RA and osteoarthritis.

## 4. Materials and Methods

### 4.1. Animals and Adjuvant Arthritis

In this experiment, Lewis’s male rats were purchased from the Department of Toxicology and Laboratory Animal Breeding, Centre of Experimental Medicine, SAS, Dobrá Voda, Slovak Republic (SK CH 24011). Rats were kept in quarantine for 7 days following the housing of the animals. The animals were kept in a 12 h/12 h dark/light cycle and were provided unfettered access to a regular feed and tap water. The EU Convention for the Protection of Vertebrate Animals Used for Experimental and Other Purposes was fulfilled in terms of animal housing. The State Veterinary and Food Administration of the Slovak Republic, Bratislava (3144/16-221/3), and the Ethics Committee of the Centre of Experimental Medicine SAS in Bratislava, Slovakia, approved the protocol for this experiment. We have applied the 3Rs (replacement, reduction, and refinement) concept in compliance with Directive 2010/63/EU [61]. Currently, scientific guidelines at the European and worldwide levels of the ICH (International Cooperation on Harmonization of Technical Requirements) incorporate the use of the 3Rs [62]; likewise, we followed these current scientific guidelines in our in vivo experiments.

### 4.2. Induction of Adjuvant Arthritis in Lewis Rats

Adult male Lewis rats were used to establish the adjuvant arthritis model (AA). It is a well-known model of inflammation [13,63] that is frequently used by our scientific team [13,15,16]. The animal model used in this study was previously used by other researchers [17,64]. A 0.1 mL suspension of heat-inactivated Mycobacterium butyricum (Difco, Detroit, MI, USA) with incomplete Freund’s adjuvants (Thermo Fisher Scientific, Waltham, MA, USA) at a dosage of 12 mg/mL was injected into experimental rats’ tail base (0.1 mL) at beginning of the experiments. The suspension was administered via subcutaneous injection to all animal except the healthy control. All animals were orally treated by gavage once daily for 28 days, and the doses were specifically calculated based on the body weight (b.w.) of each animal. The animals were weighed once a day before the administration of each substance tested.

### 4.3. The Design of the Preliminary Experiment

The rats were randomly assigned to the six experimental groups. Group 1 served as a healthy control. The AA group without treatment was the second. The AA rats in the four treated groups were handled in accordance with the research design shown below in Table 1. The number of animals in the groups was 5–8.

Healthy controls and AA animals received tap water daily per os, while all treated AA groups received the experimental treatment as per the study design (Table 1). Hyaluronan (HA) (0.99 MDa—SHA and 1.73 MDa—VHA) was purchased from company Contipro a.s., Czech Republic. We dissolved the HA molecules in sterile saline solution. In this study, the HA doses of 0.5 mg/kg/day and 5 mg/kg/day used are close to those used in an experimental setting by Cilaker Micili et al. (2023) [65].

### 4.4. The Design of the Combination Therapy Experiment—The Pivotal Experiment

Rats were randomly assigned to the seven experimental groups. Group 1 served as a healthy control. The AA group without treatment was the second. The AA rats in the five treated groups were handled in accordance with the research design shown below in Table 2. The number of animals in groups was 6–8.

Healthy controls and AA animals received tap water daily per os, while all treated AA groups received the experimental treatment as per the study design (Table 2). As in preliminary experiment, HA samples were received as gift from company Contipro a.s., Czech Republic. Additionally, one AA group received MTX monotherapy, and two AA groups were administered methotrexate (MTX; EBEWE, Unterach am Attersee, Austria) in combination treatment with HA. The tested substances and MTX were administered orally (via gastric tube) throughout the entire experiment; MTX was used at a subtherapeutic dose of 0.3 mg/kg (two times per week), and all HA forms and doses were administered daily (Table 2). The animals were anesthetized with Zoletil administered with Xylariem, and blood samples were obtained. For this procedure, a pre-heparinized pipette (10 µL) was introduced into retro-orbital sinus of the rats on experimental day 14 and 21. The obtained blood was centrifuged (using an Eppendorf 5702 R centrifuge, Hamburg, Germany) at 3000 r.p.m. for 15 min and at a temperature of 4 °C. After a time-out section, the blood tubes were removed from the centrifuge, and the resulting supernatant was stored at a temperature of −80 °C in a freezer for the evaluation of biochemical parameters. On the last experimental day (day 28), the animals were put under deep anaesthesia (Zoletil + Xylariem), and the blood was collected by heart puncture with a syringe containing heparin. The blood was centrifugated as described above. Individual organs and tissues were dissected and weighed on analytical scales. They were then divided according to the respective weights; then, they were frozen with liquid nitrogen and stored at −80 °C.

### 4.5. Evaluation of Experimental AA

Biometric parameters were evaluated to assess arthritis, following the methodology from our previous studies [9,15]. Paw edema volume was measured on days 14, 21, and 28 after immunization. The biometric parameter change in hind-paw volume (ChPV) was measured using a water plethysmometer (UGO BASILE, Gemonio, Italy). The results were presented as the average percentage increase, representing the hind-paw volume of each rat relative to the HPV measured on day 1. The HPV on the selected day was divided by the HPV on day 1 and then expressed as a percentage, as follows:

([Day n]/[Day 1]) × 100 − 100 = value [%].

Every day, the animals’ body weight was recorded to guarantee accurate dosage, and the following formula was used to determine the changes in body weight (ChBW) on days 14, 21, and 28:

[Day n] − [Day 1] = value [g].

### 4.6. Measurement of Gamma-Glutamyl Transferase Activity in the Spleen and Hind-Paw Joint Tissue

Like in our previous work [16], we measured the activity of gamma-glutamyl transferase (GGT) in the hind-paw joint and spleen tissue homogenates on day 21 applying the method of Orlowski and Meister (1970) [66], which was modified by Ondrejickova et al. (1993) [67]. Tissues were homogenized for one minute at 0 °C using an Ultra Turax TP 18/10. A phosphate buffer (pH 8.1, 2.6 mM NaH_2_PO_4_, 50 mM Na_2_HPO_4_, 15 mM EDTA, and 68 mM NaCl) was used for homogenization. The biological substrates were then diluted to final concentrations of 2.5 mM and 12.6 mM, respectively, in 65% isopropyl alcohol. The substrates used were 44 mM methionine and 8.7 mM L-glutamyl-p-nitroanilide. After incubating the samples for one hour at 37 °C, 2.3 mL of cold methanol was added to stop the reaction. The tubes were then centrifuged for 20 min using an Eppendorf centrifuge (rotor radius = 7 cm, 5000 rpm) at a centrifugal force of 1957× *g*. The absorbance of the supernatant (p-nitroaniline product) was measured at 406 nm with a Specord 40 spectrophotometer (Analytikjena, Jena, Germany). Blank solutions with and without the acceptor or substrate were also used. Activity was determined by measuring the absorbance and applying a calibration coefficient.

### 4.7. Measurement of IL-17A

In accordance with our previous methodology [13,68], blood was extracted on day 14 from the rat’s retroorbital sinus and centrifuged. The plasma was then stored at −80 °C. On the 28th day of the experiment, blood was collected via heart puncture under deep anesthesia for analysis. An enzyme-linked immunosorbent assay (ELISA) kit (R&D Systems, Minneapolis, MN, USA) was used to measure the IL-17A concentrations in the plasma samples, following the manufacturer’s instructions.

### 4.8. Activity of Superoxide Dismutase and Glutathione Peroxidase in Erythrocytes and Measurement of Lipid Peroxidation in Plasma Samples

#### 4.8.1. Hemolysate Preparation and Determination of Hemoglobin

Diluted 1:3 (*v*/*v*) with ice-cold distilled water, washed erythrocytes were then incubated for 15 min. After that, the hemolysate was kept at −20 °C until a further examination. Using the Drabkin technique [69], the hemoglobin concentration in the hemolysate was determined and expressed in g/L.

#### 4.8.2. The Activity of Antioxidant Enzymes

Following the manufacturer’s instructions, a commercial kit (Sigma-Aldrich, No. 19160, St. Louis, MO, USA) was used to assay the superoxide dismutase (SOD) activity in erythrocyte hemolysates. Units per milligram of hemoglobin (U/mg Hb) were used to report the activity.

The activity level of glutathione peroxidase (GPx) in erythrocyte hemolysates was measured using the glutathione peroxidase activity kit (Cayman Chemical, No. 703102, Ann Arbor, MI, USA), following the manufacturer’s instructions. The activity was expressed as micromoles of substrate converted per second per milligram of hemoglobin (µkat/mg Hb).

#### 4.8.3. Marker of Oxidative Stress in Plasma

The concentration of lipoperoxides in serum was determined according to the method outlined by El-Saadani et al. [70]. This technique makes use of peroxides’ capacity to convert iodide (I^−^) to iodine (I_2_). Triiodide (I_3_^−^), which has an absorbance maximum at 365 nm, is created when the generated iodine combines with excess iodide. The concentration of lipoperoxide was stated in nmol/mL.

### 4.9. Statistical Analysis of the Experimental Results

The experimental data were expressed using the arithmetic mean and ± standard error of the mean (SEM). The GraphPad InStat3 software was used to identify the experimental groups’ statistically significant variations. Any significant changes between the treatment groups of animals (AA-SHA, AA-5SHA, AA-VHA, AA-5VHA, AA-MTX, AA-MTX + SHA, and AA-MTX + VHA) and the control animals (HC), untreated animals (AA), and animals were assessed using ANOVA. When there were notable differences between the groups, the post hoc test (Tukey–Kramer) was applied. The degrees of significance were as follows after the post hoc screening: not significant (*p* > 0.05), significant (*p* ≤ 0.05), very significant (*p* ≤ 0.01), and extremely significant (*p* ≤ 0.001). Information on the particular symbol of significance is provided in the legend beneath each table and graph.

### 4.10. Limitations of the Study

Animal models can only capture certain aspects of the complex pathology of diseases like rheumatoid arthritis (RA). A challenge arises from the fact that human RA develops due to a combination of genetic predisposition and environmental factors, making it difficult to fully replicate in controlled experimental settings. Given that inbred animals are exposed to a specific arthritogenic stimulus, they may not fully represent the multifaceted nature of RA. Therefore, it is the researcher’s responsibility to assess whether a given model effectively mimics a relevant aspect of human pathology and to interpret the findings accordingly. One advantage of animal models is their ability to eliminate confounding factors that often complicate the evaluation of therapeutic efficacy in human patients. Additionally, adjuvant arthritis offers a useful feature in its non-specific immune activation, which leads to joint inflammation [71].

Similar to the unclear origins of RA, the precise role of mineral oil and mycobacterial components in complete Freund’s adjuvant (CFA) in triggering joint disease remains uncertain. The activation process of the adjuvant can cause systemic macrophage overactivation, promoting the expansion of autoimmune T and B cell populations. These immune cells may infiltrate joints and stimulate the production of autoantibodies against heat shock proteins (Hsps) and cartilage-specific antigens. Furthermore, cytokines secreted by macrophages directly influence chondrocytes and synovial cells, leading to abnormal matrix component synthesis, irregular adhesion molecule expression, upregulation of MHC class II antigens, and synovial hypertrophy. Another limitation of adjuvant arthritis as a model for RA is its rapid disease onset, occurring within 16–19 days or even sooner, as observed in this study (around day 14). This differs significantly from the gradual and chronic progression characteristic of RA. However, in pharmaceutical research, where financial constraints often influence model selection, the early onset of disease is considered advantageous for accelerating drug development [72]. The study is also limited by the sample size, as well as by considerations related to the 3R (replacement, reduction, and refinement) principles. Additionally, the pharmacokinetics of the tested substances and their potential interactions may have influenced certain observed effects. Furthermore, a significant limitation is the lack of molecular-level analysis to elucidate the precise mechanisms of action.

## 5. Conclusions

The overall efficacy ranking for the pivotal experiment could be drawn as follows: MTX ≈ MTX + VHA < MTX + SHA. This highlights the superior therapeutic effect of SHA when combined with MTX

We observed that the combination of MTX and HA with a molecular weight of 0.99 MDa (SHA) had the following therapeutic advantages: it significantly boosted the weight gain in AA animals on day 28, even compared to MTX monotherapy, which was without significance, and it was the only treatment effective in reducing GGT activity levels in both the spleen and joints on day 28.

SHA monotherapy significantly reduced IL-17A levels on day 28, while both tested HA variants lowered plasma LPx concentrations compared to untreated AA rats. The suitability of oral HA administration was shown.

Oral administration is well tolerated by the patient and can enhance treatment by MTX, highlighting the anti-inflammatory potential of SHA; it may be an appropriate adjunct therapy with other RA treatments. Further research needs to be carried out to elucidate the exact mechanism of oral SHA.

## Figures and Tables

**Table 1:** The design of the preliminary experimental groups.

Groups	Treatments	Doses
Healthy control (HC)	Vehicle	0.5 mL
Adjuvant arthritis (AA)	Vehicle	0.5 mL
SHA	AA + HA, molecular weight of 0.99 MDa	0.5 mg/kg/day
5 SHA	AA + HA, molecular weight of 0.99 MDa	5 mg/kg/day
VHA	AA + HA, molecular weights of 1.73 MDa	0.5 mg/kg/day
5 VHA	AA + HA, molecular weights of 1.73 MDa	5 mg/kg/day

**Table 2:** The design of the combination therapy experimental groups.

Groups	Treatments	Doses
Healthy control (HC)	Vehicle	0.5 mL
Untreated adjuvant arthritis (AA)	Vehicle	0.5 mL
AA-SHA	AA + HA, molecular weight of 0.99 MDa	0.5 mg/kg/day
AA-VHA	AA + HA, molecular weights of 1.73 MDa	0.5 mg/kg/day
AA-MTX	Methotrexate (MTX)	0.3 mg/kg/twice per week
AA-MTX + SHA	AA + HA, molecular weight of 0.99 MDa + MTX	0.5 mg/kg/day + 0.3 mg/kg/twice per week
AA-MTX + VHA	AA + HA, molecular weight of 1.73 MDa + MTX	0.5 mg/kg/day + 0.3 mg/kg/twice per week

## Data Availability

All raw data used are available at the link below. https://figshare.com/articles/figure/Hyaluronan_Raw_data_xlsx/27297036?file=49971996 (accessed date on 24 October 2024).
